# Effects of exercise after oesophagectomy on body composition and adequacy of energy and protein intake: PERFECT multicentre randomized controlled trial

**DOI:** 10.1093/bjsopen/zrad057

**Published:** 2023-08-01

**Authors:** Anouk Hiensch, Elles Steenhagen, Jonna K van Vulpen, Jelle P Ruurda, Grard A P Nieuwenhuijzen, Ewout A Kouwenhoven, Richard P R Groenendijk, Donald L van der Peet, Camiel Rosman, Bas P L Wijnhoven, Mark I van Berge Henegouwen, Hanneke W M van Laarhoven, Richard van Hillegersberg, Peter D Siersema, Anne M May

**Affiliations:** Julius Center for Health Sciences and Primary Care, University Medical Center Utrecht, Utrecht University, Utrecht, The Netherlands; Department of Dietetics, University Medical Center Utrecht, Utrecht, The Netherlands; Department of Radiation Oncology, University Medical Center Utrecht, Utrecht, The Netherlands; Department of Surgery, University Medical Center Utrecht, Utrecht, The Netherlands; Department of Surgery, Catharina Hospital, Eindhoven, The Netherlands; Department of Surgery, ZGT Hospital, Almelo, The Netherlands; Department of Surgery, IJsselland Hospital, Capelle a/d IJssel, The Netherlands; Department of Surgery, Amsterdam UMC location VUmc, Amsterdam, The Netherlands; Department of Surgery, Radboud University Medical Center, Nijmegen, The Netherlands; Department of Surgery, Erasmus Medical Center, Rotterdam, The Netherlands; Department of Surgery, Amsterdam UMC location University of Amsterdam, Amsterdam, The Netherlands; Cancer Center Amsterdam, Cancer Treatment and Quality of Life, Amsterdam, The Netherlands; Cancer Center Amsterdam, Cancer Treatment and Quality of Life, Amsterdam, The Netherlands; Department of Medical Oncology, Amsterdam UMC location University of Amsterdam, Amsterdam, The Netherlands; Department of Surgery, University Medical Center Utrecht, Utrecht, The Netherlands; Department of Gastroenterology and Hepatology, Radboud University Medical Center, Nijmegen, The Netherlands; Julius Center for Health Sciences and Primary Care, University Medical Center Utrecht, Utrecht University, Utrecht, The Netherlands

## Introduction

Patients with localized oesophageal cancer may experience long-lasting symptoms following cancer treatment (that is often neoadjuvant chemo(radio)therapy followed by oesophagectomy)^1,2^, resulting in suboptimal intake of nutrients in the first postoperative year^3^. This might cause persistent weight and muscle loss and a progressive decline in nutritional status^4–9^. Strategies to counteract weight and muscle loss involve exercise and nutritional interventions^10,11^. However, specific recommendations for nutritional interventions during oncological exercise programmes are lacking^12^.

In the randomized Physical ExeRcise Following Oesophageal Cancer Treatment (PERFECT) study, a 12-week supervised exercise programme was demonstrated to be safe and feasible after oesophagectomy and effective in terms of improved quality of life (QoL) (primary outcome), role functioning, and cardiorespiratory fitness^13^. As part of the secondary analysis of the PERFECT study, the aim is to assess whether or not participants in the PERFECT study meet their energy and protein requirements and investigate exercise effects on body composition, malnutrition risk, and energy expenditure. Getting more insight into this would be of great importance for optimal nutritional strategies during the recovery phase.

## Methods

### Setting and participants

The PERFECT study is a multicentre randomized controlled trial performed in nine Dutch hospitals between 2015 and 2019. The design of the PERFECT study has been published previously^14^. The trial was registered on 19 January 2015 in the Dutch Trial Register (NTR 5045) (https://trialsearch.who.int/Trial2.aspx? TrialID = NTR5045). See *Text S1* for inclusion and exclusion criteria. The study was approved by the Medical Ethics Committee of the University Medical Center (UMC) Utrecht and the local Ethical Boards of participating hospitals.

After signing written informed consent and completing baseline measurements, participants were randomly allocated to a 12-week supervised exercise intervention (EX) or usual care group (UC).

### Dietetic study

Patients included in the UMC Utrecht were asked to participate in optional dietetics measurements. During these measurements, resting energy expenditure (REE), body composition, and nutritional status were measured.

Additional informed consent was obtained before baseline testing and randomization.

### Intervention

The exercise intervention consisted of a 12-week supervised, individualized, combined aerobic and resistance exercise programme, in addition to UC. Details of the exercise programme have been published elsewhere and are summarized in *Text S1*^14^.

### Outcome measures

*Table 1* provides an overview of all outcome measures of the PERFECT study, which are of interest for the current secondary analysis. Detailed information about all outcome measures can be found in *Text S1*.

**Table 1:** Outcome assessment in all PERFECT participants and in the dietetic subgroup

Outcome assessment	All PERFECT participants	Dietetic subgroup
**Dietary intake**		
Energy intake	X	X
Protein intake	X	X
**Anthropometry**		
Weight	X	X
Height	X	X
**Body composition**		
Fat mass (kg), fat mass index (kg/m^2^) Fat free mass (kg), fat free mass index (kg/m^2^)		X
		X
**Resting energy expenditure**		
Estimated (WHO-formula)	X	X
Measured (indirect calorimetry)		X
Oxygen consumption (VO_2_), carbon dioxide production (VCO_2_), and respiratory quotient (RQ)		X
**PG-SGA**		
Risk for malnutrition	X	X
Nutritional status		X

PG-SGA, Patient Generated Subjective Global Assessment.

### Statistical analysis

A paired samples *t* test was performed to assess adequacy of dietary intake. All outcomes were analysed as between-group differences in outcomes using intention-to-treat analysis of covariance (ANCOVA). Detailed information can be found in *Text S1*.

## Results

### Participants

In total, 79 of all 120 PERFECT participants were invited to participate in the optional dietetic measurements and 37 participants agreed. In the dietetic study, five participants were lost to follow-up (EX: three of 19, UC: two of 18) (*Fig. S1*).

Baseline and nutritional characteristics of all PERFECT participants and participants in the dietetic study are shown in *Table S1* and *Table S2* respectively.

### All participants (*n* = 120)

#### Dietary intake

At baseline (3 (2–7) months postoesophagectomy (median, interquartile range (i.q.r.)), participants had a mean energy and protein intake of 2413 kcal/day (s.d. = 640) and 94 gram/day (s.d. = 28) respectively. No clinically relevant changes in energy and protein intake were observed in either group during the intervention period. Postintervention, energy and protein intake were comparable between groups (*Table 2*). At baseline, 63.2 per cent and 37.6 per cent of all participants had an adequate energy and protein intake respectively (*Tables S3–S5)*. At 12 weeks these percentages were 66.0 per cent and 33.3 per cent respectively.

**Table 2:** Effects of the PERFECT exercise intervention on weight, risk for malnutrition and dietary intake among all participants in the PERFECT study (*n* = 120) and effects of the PERFECT exercise intervention on weight, body composition, nutritional status, resting energy expenditure, and dietary intake in the dietetic subgroup (*n* = 37)

		Baseline	Baseline to 12 weeks (post-intervention)		
		Mean(s.d.)	Within-group differences	Between-group differences	Effect size
			Mean [95% c.i.]	Mean [95% c.i.]	
**All PERFECT participants (*n* = 120)**					
**Weight (kg)**	EX	76.1(12.5)	−1.16 [−1.95, −0.37]*	−1.19 [−2.48, 0.10]	0.09
	UC	78.2(13.3)	−0.63 [−1.41, 0.15]	Reference	
**Risk for malnutrition**	EX	7.4(5.2)	−1.88 [−3.21, −0.55]*	−0.17 [−2.12, 1.79]	0.03
	UC	7.4(5.1)	−0.64 [−1.96, 0.68]	Reference	
**Dietary intake**					
Energy intake kcal/kg/day	EX	32.5(8.9)	0.53 [0.20, 0.87]*	0.36 [−0.17, 0.89]	0.04
	UC	31.1(8.3)	0.17 [−0.23, 0.56]	Reference	
Protein intake gram/kg/day	EX	1.27(0.37)	0.02 [−0.08, 0.11]	0.01 [−0.11, 0.13]	0.03
	UC	1.20(0.36)	0.05 [−0.04, 0.14]	Reference	
**Dietetic subgroup (*n* = 37)**					
**Dietary intake**					
Energy intake (kcal/kg/day)	EX	33.1(10.1)	0.68 [−0.07, 1.42]	0.41 [−0.72, 1.54]	0.05
	UC	29.2(8.0)	0.15 [−0.48, 0.78]	Reference	
Protein intake (gram/kg/day)	EX	1.3(0.37)	−0.11 [−0.23, 0.01]	−0.13 [−0.35, 0.09]	0.37
	UC	1.1(0.34)	0.10 [−0.08, 0.29]	Reference	
Protein intake (gram/kg FFM/day)	EX	1.7(0.41)	−0.18 [−0.33, −0.02]٭	−0.17 [−0.47, 0.13]	0.41
	UC	1.5(0.43)	0.12 [−0.14, 0.38]	Reference	
**Weight (kg)**	EX	73.5(13.0)	−1.68 [−3.81, 0.45]	−1.51 [−4.56, 1.54]	0.12
	UC	78.0(12.2)	−0.29 [−1.90, 1.32]	Reference	
**Body composition**					
FFMI (kg/m²)	EX	18.2(2.1)	−0.04 [−0.41, 0.33]	−0.11 [−0.67, 0.45]	0.05
	UC	18.2(2.1)	0.09 [−0.25, 0.43]	Reference	
FMI (kg/m²)	EX	6.2(1.9)	−0.51 [−0.94, −0.09]*	−0.36 [−0.91, 0.18]	0.20
	UC	6.4(1.7)	−0.20 [−0.61, 0.22]	Reference	
**Resting energy expenditure**					
REE measured (kcal/day)	EX	1597(237)	66.19 [−2.95, 135.33]	76.46 [−31.92, 184.84]	0.30
	UC	1777(268)	−85.25 [−161.23, −9.27]*	Reference	
REE measured (kcal/kg/day)	EX	22.0(2.9)	1.38 [0.50, 2.26]*	1.62 [0.42, 2.81]٭	0.62
	UC	22.6(2.3)	−0.96 [−1.95, 0.04]	Reference	
REE measured (kcal/kg FFM/day)	EX	28.5(2.9)	1.29 [0.14, 2.44]*	1.17 [−0.39, 2.74] Reference	0.39
	UC	30.1(3.1)	−1.64 [−3.22, −0.07]*		
RQ, measured REE	EX	0.80(0.07)	−0.03 [−0.07, −0.003]*	−0.06 [−1.1, −0.01]٭	1.10
	UC	0.77(0.04)	0.05 [0.01, 0.09]*	Reference	
VO_2_ (l/min), measured REE	EX	232.05(35.71)	11.44 [0.90, 21.98]*	14.60 [−2.36, 31.56]	0.39
	UC	260.17(40.04)	−15.38 [−27.42, −3.34]*	Reference	
VCO_2_ (l/min), measured REE	EX	185.37(27.34)	1.31 [−7.59, 10.22]	−5.13 [−18.44, 8.17]	0.18
	UC	199.61(28.24)	0.88 [−8.43, 10.18]	Reference	
**Nutritional status (PG**−**SGA)**					
Risk for malnutrition	EX	8.6(4.7)	−1.94 [−4.23, 0.35]	−2.22 [−5.85, 1.41]	0.47
	UC	6.1(4.7)	1.61 [−1.39, 4.61]	Reference	
Nutritional status score	EX	10.3(4.9)	−2.56 [−5.05, −0.08]*	−3.73 [−7.60, 0.15]	0.78
	UC	7.3(4.7)	2.13 [−1.10, 5.36]	Reference	

c.i., confidence interval; ES, effect size; EX, exercise group; s.d., standard deviation; UC, usual care group; FFM, fat free mass; FFMI, fat free mass index; FMI, fat mass index; REE, resting energy expenditure; RQ, respiratory quotient (VCO_2_/VO_2_); VO_2_, oxygen consumption; VCO_2_, carbon dioxide production; PG−SGA, Patient-Generated Subjective Global Assessment. Effect sizes <0.2 indicate ‘no difference’, effect sizes of 0.2–0.5 indicate ‘small differences’, effect sizes of 0.5–0.8 indicate ‘medium differences’ and effect sizes ≥0.8 indicate ‘large differences’.

#### Weight and risk for malnutrition

EX had a non-significant lower weight at 12 weeks compared with UC (−1.19 kg, 95 per cent c.i. −2.48 to 0.10, ES = 0.09) (*Table 2*). Malnutrition risk declined within EX, but no significant difference was observed when compared with UC.

### Dietetic subgroup (*n* = 37)

#### Dietary intake

Postintervention, no significant differences in energy and protein intake between EX and UC were observed (*Table 2*). EX had a lower protein intake per kg weight per day (−0.11 g per kg per day, 95 per cent c.i. −0.23 to 0.01) and per kg fat free mass (FFM) per day (−0.18 g per kg FFM per day, 95 per cent c.i. –0.33 to −0.02) post-intervention compared with baseline. Similarly, a decline in adequacy of protein intake in g/FFM was observed from baseline to 12 weeks in EX (from 57.9 per cent to 25.0 per cent) (*Tables S5* and *S6*). An adequate energy intake at baseline and 12 weeks respectively, was found in 52.6 per cent and 43.8 per cent of EX and 58.8 per cent and 43.8 per cent of UC (*Table S4*).

#### Weight and body composition

Postintervention, the between-group difference in weight was −1.51 kg (95 per cent c.i. −4.56 to 1.54, ES = 0.12). This decline in weight was mainly due to a decreased fat mass (FM) index from baseline to 12 weeks in EX (−0.51, 95 per cent c.i. −0.94 to −0.09), whereas the FFM index remained stable over time (*Table 2)*. No statistically significant between-group differences in FM indices were observed postintervention (−0.36, 95 per cent c.i. −0.91 to 0.18, ES = 0.20).

#### Measured resting energy expenditure

Postintervention, EX had a significantly higher measured REE (mREE) per kg weight (1.62 kcal/kg, 95 per cent c.i. 0.42 to 2.81, ES = 0.62), mREE per kg FFM (1.17 kcal/kg FFM, 95 per cent c.i. −0.39 to 2.74, ES = 0.39), measured oxygen consumption (VO_2_) (14.60 ml/min, 95 per cent c.i. −2.36 to 31.56, ES = 0.39), and a significantly lower respiratory quotient (RQ) (−0.06 ml/min, 95 per cent c.i. –1.1 to −0.01, ES = 1.10) compared with UC (*Table 2*).

#### Risk of malnutrition and nutritional status

Postintervention, risk of malnutrition tended to be lower for EX compared with UC (−2.22, 95 per cent c.i. −5.85 to 1.41, ES = 0.47). Similarly, postintervention nutritional status tended to be better for EX compared with UC (−3.73, 95 per cent c.i. −7.60 to 0.15, ES = 0.78) (*Table 2)*.

## Discussion

This study showed that the majority of patients after oesophagectomy do not meet estimated protein requirements, especially when increasing physical activity levels as part of an exercise programme. Only slightly more than half of all participants meet the estimated energy requirements. These numbers were even lower when energy intake was compared with daily energy requirements calculated using the mREE. Measurement of REE is recommended to personalize energy needs^15^.

Patients participating in the PERFECT exercise programme were observed to lose more weight compared with controls, which seemed to be mainly loss of FM. FFM, which is commonly used as a proxy for skeletal muscle mass, remained stable over time in the exercise group. Since the exercise programme included progressive resistance training, an increase in muscle mass was expected. This counterintuitive finding could potentially be explained by the decreased protein intake in the exercise group. These findings suggest the need for a structured nutritional intervention, in addition to an exercise programme, which likely will result in larger effects of the exercise programme^16,17^. This might be of even greater importance for patients receiving immunotherapy after surgery in order to be able to complete this treatment^18^.

Exercise had a positive effect on the risk of being malnourished, while energy and protein intake was inadequate and patients participating in the exercise programme lost weight. The questionnaire used to measure risk of malnutrition consists of four domains: weight, food intake, nutrition impact symptoms, and activities and function. The scores for these particular domains remained fairly stable over time and were not different between groups, except for the nutrition impact symptom domain (*Table S2*). This suggests that the PERFECT exercise programme reduces patients’ relevant symptoms, leading to improvements in the patients’ nutritional status^13^.

This study has several limitations. Only half of the PERFECT participants decided to participate in the dietetic study, hampering the generalizability of the findings. Secondary outcomes are reported here, for which the study was not powered. Due to self-selection, patients who participated in the dietetic study had a relatively higher risk of malnutrition at baseline (43.2 per cent) compared with all participants (32.5 per cent). Finally, no data were available regarding the number of consultations with a dietitian during the study period and the specific recommendations that were given during this encounter.

The present study shows that patients in the first year after oesophagectomy are at risk of a suboptimal energy and protein intake, especially when increasing physical activity levels as part of an exercise programme. The results suggest that exercise has a small positive effect on the risk of being malnourished, and weight loss was mainly loss of FM and not FFM. These findings highlight the need for specific nutritional recommendations during oncological exercise programmes.

## Supplementary Material

zrad057_Supplementary_Data

## Data Availability

Data supporting the findings are available from the corresponding author upon reasonable request.
