# Supplementary material for: Effects of exercise after oesophagectomy on body composition and adequacy of energy and protein intake: PERFECT multicentre randomized controlled trial
Source: BJS Open. 2023 Aug 1;7(4):zrad057. doi: 10.1093/bjsopen/zrad057 (PMC10392959; doi:10.1093/bjsopen/zrad057)
Supplement: zrad057_Supplementary_Data [file zrad057_supplementary_data.docx]

**Effects of exercise after oesophagectomy on body composition and adequacy of energy and protein intake: the PERFECT multicenter randomized controlled trial**

Anouk Hiensch, PhD^1*^, Elles Steenhagen RD^2*^, Jonna K van Vulpen MD, PhD^,3^, Jelle P. Ruurda MD, PhD^4^, Grard A.P. Nieuwenhuijzen MD, PhD^5^, Ewout A. Kouwenhoven MD, PhD^6^, Richard P.R. Groenendijk MD, PhD^7^, Donald L. van der Peet MD, PhD^8^, Camiel Rosman MD, PhD^9^, Bas P.L Wijnhoven MD, PhD^10^, Mark I. van Berge Henegouwen MD, PhD^11,12^, Hanneke W.M. van Laarhoven MD, PhD^12,13^, Richard van Hillegersberg MD, PhD^4^, Peter D. Siersema MD, PhD^14^, Anne M. May, PhD^1^

*Shared first authors

^1^ Julius Center for Health Sciences and Primary Care, University Medical Center Utrecht, Utrecht University, Utrecht, The Netherlands

^2^ Department of Dietetics, University Medical Center Utrecht, Utrecht, The Netherlands

³ Department of Radiation Oncology, University Medical Center Utrecht, Utrecht, The Netherlands

^4^ Department of Surgery, University Medical Center Utrecht, Utrecht, The Netherlands

^5^ Department of Surgery, Catharina Hospital, Eindhoven, The Netherlands

^6^ Department of Surgery, ZGT Hospital, Almelo, The Netherlands

^7^ Department of Surgery, IJsselland Hospital, Capelle a/d IJssel, The Netherlands

^8^ Department of Surgery, Amsterdam UMC location VUmc, Amsterdam, The Netherlands

^9^ Department of Surgery, Radboud University Medical Center, Nijmegen, The Netherlands

^10^ Department of Surgery, Erasmus Medical Center, Rotterdam, The Netherlands

^11^ Department of Surgery, Amsterdam UMC location University of Amsterdam, Amsterdam, The Netherlands

^12^ Cancer Center Amsterdam, Cancer Treatment and Quality of Life, Amsterdam, The Netherlands

¹^3^ Department of Medical Oncology, Amsterdam UMC location University of Amsterdam, Amsterdam, The Netherlands

¹^4^ Department of Gastroenterology and Hepatology, Radboud university medical center, Nijmegen, The Netherlands

**Corresponding author.** Prof. dr. Anne May, Julius Centre for Health Sciences and Primary Care, University Medical Centre Utrecht, P.O. Box 85500, 3508 GA Utrecht, The Netherlands (e-mail: [A.M.May@umcutrecht.nl](mailto:A.M.May@umcutrecht.nl))

**Supplementary Materials - Index**

| **Supplementary Text, Figures and Tables** |  |
| --- | --- |
| **Supplementary Text** | *pag. 3* |
| **Table S1 -** Baseline characteristics of all participants in the PERFECT study and participants in the dietetic study. | *pag. 9* |
| **Table S2 -** Nutritional characteristics of all participants in the PERFECT study and participants in the dietetic study. | *pag. 10* |
| **Table S3** - Difference between energy intake and measured and estimated total energy expenditure. | *pag. 11* |
| **Table S4** - Adequacy of energy intake. | *pag. 12* |
| **Table S5** - Difference between protein intake and protein  recommendations (g/kg and g/FFM). | *pag. 13* |
| **Table S6** - Adequacy of protein intake. | *pag. 14* |
| **Figure S1 -** Flow of participants in the PERFECT study and dietetic study. | *pag. 15* |

**Supplementary Text S1**

**Methods**

The CONSORT guidelines were used to facilitate reporting of the results of the PERFECT study and the study was conducted in accordance with the Declaration of Helsinki.

*In- and exclusion criteria*

Eligible patients were invited to participate by their medical specialist or oncological nurse during a regular outpatient visit. Inclusion criteria were: surgery with curative intent for newly diagnosed, histologically confirmed oesophageal cancer; 4-52 weeks after hospital discharge following surgery; 18 years or older; able to read and understand the Dutch language; able to walk ≥60 meters; physically inactive (≤150 min per week of moderate-vigorous exercise) and a Karnofsky Performance Status ≥60. Exclusion criteria were: presence of metastatic oesophageal cancer based on CT-imaging prior to surgery; non-radical resection; contra-indications for physical activity (as assessed through the Revised Physical Activity Readiness Questionnaire); and/or involvement in another comparable supervised exercise program.

For participation in the dietetic study one additional exclusion criterion was formulated: the presence of a pacemaker.

*Intervention*

The exercise program included two combined aerobic and resistance training exercise sessions of one hour per week, supervised by an outpatient (oncology) physiotherapist close to the participant’s home address. Adherence and compliance with the supervised exercise program was above 90%. In addition, participants were encouraged to be physically active for at least 30 min/day on all remaining days of the week as recommended by the WCRF/AIC guidelines for cancer survivors. Participants randomized to the control group received usual care and were asked to maintain their habitual physical activity pattern. After completion of the study, the control group was offered an exercise advice.

*Outcome measures*

Table 1 provides an overview of all outcome measures of the PERFECT study, which are of interest for the current secondary analysis. These outcomes are assessed in all participants and in the dietetic subgroup, both at baseline and post-intervention (12 weeks). The measurements were performed in a fasted state; in the 8 hours prior to the measurements, patients were instructed to abstain from smoking, food and drinks, except for water 2 hours prior to the measurements.

*Dietary intake*Dietary intake was assessed using a 3-day food diary, which was filled out on two non-consecutive weekdays and one weekend day. Diaries were checked by a trained researcher or dietitian and were, in case of incompleteness, completed during a telephone call with the participant. Food records were coded and analysed for energy and protein intake by trained dietitians, using the Dutch Food Composition Database (Nevo 2013, National Institute for Public Health and the Environment (RIVM), The Netherlands).
 *Energy and protein requirements and adequacy of dietary intake*

For all PERFECT participants, daily energy requirements were estimated using the WHO equation (1985)^1^, adjusted for physical activity levels. For the dietetic subgroup, daily energy requirements were calculated using the measured resting energy expenditure (REE), adjusted for physical activity levels. In this study, a factor of 1.5 was used to reflect increased physical activity levels.^2,3^

Protein requirements were calculated as 1.5 gram protein per kg of ideal body weight for all PERFECT participants and as 1.9 gram protein per kg of fat free mass (FFM) for the dietetic subgroup.^4^ This is in line with the ESPEN recommendations, which recommend that the protein intake should be above 1 g/kg/day and, if possible up to 1.5 g/kg/day.^5^ We hypothesize that the higher end of this range should be recommended to cancer survivors who take part in an exercise program with the aim to maintain or improve muscle mass. For the ideal body weight, the actual weight was used for participants with a BMI between 20 and 27.5. For those with a BMI under 20, the hypothetical body weight at BMI 20 was used, whereas the hypothetical body weight at BMI 27.5 was used for those with a BMI over 27.5.^6–8^ Daily dietary intake was considered adequate if the mean intake was greater than 90% of estimated requirements for energy and protein.

*Anthropometry*Height and body weight were measured, wearing light clothes and no shoes. Body Mass Index was calculated as weight (kg)/height (m²).

*Body composition*

In the dietetic subgroup, FFM and fat mass (FM) were measured using a whole-body multifrequency BIA device (Bodystat Quadscan 4000; Bodystat Ltd, Douglas, UK), according to institutional standard operating procedures. Participants were asked to empty their bladder ≥30 min before the procedure. FFM was calculated using the equation of Kyle.^9^ FM was calculated by subtracting FFM from total body weight. The FFM and FM index were calculated as FFM (in kg)/height (m²) and FM (in kg)/height (m²). A low FFM Index (FFMI) was defined as <16.7 kg/m² for men and <14.6 kg/m² for women.^10^

A low FFMI was

defined as ≤14.6 kg/m

2

for women and ≤16.7 for men

A low FFMI was

defined as ≤14.6 kg/m

2

for women and ≤16.7 for men

A low FFMI was

defined as ≤14.6 kg/m

2

for women and ≤16.7 for men

A low FFMI was

defined as ≤14.6 kg/m

2

for women and ≤16.7 for men.

*Resting energy expenditure (REE)*

REE was estimated (pREE) for all PERFECT participants using the WHO equation (1985)^1^. In the dietetic subgroup REE was measured (mREE) using indirect calorimetry (i.e., gold standard) by a ventilated hood system (Cosmed Quark RMR; COSMED, Rome, Italy), which was calibrated before use. Oxygen consumption (VO₂, l/min), carbon dioxide production (VCO₂, l/min), and respiratory quotient (RQ, VCO₂/ VO₂) were measured in a rested state in a supine position for 30 minutes, and in a thermoneutral environment. Measured REE (mREE, kCal/d) was obtained through the Weir equation.^11^

Participants of the dietetic subgroup were classified as hypometabolic (<90%), normometabolic (90-110%), or hypermetabolic (>110%) according to the standards of Boothby and Sandiford (mREE/pREE x 100%).^12^ As body size and composition are key determinants of REE^13,14^, mREE and pREE were also divided by the participants’ body weight (kCal/kg body weight) and FFM (kCal/kg FFM).

*Risk for malnutrition and nutritional status*Risk for malnutrition was assessed for all PERFECT participants using the self-reported PG-SGA Short Form (PG-SGA SF). In accordance with the PG-SGA SF triage system, <4 points was defined as low risk, 4-8 points as medium risk, and ≥9 points as high risk for malnutrition.^15,16^ For nutritional status, the PG-SGA SF was complemented with the professional component of the PG-SGA, which was carried out by a trained dietitian for the subgroup with additional dietetic measurements only. Subsequently, the PG-SGA total score was calculated with a higher score indicating a worse nutritional status.^17^

*Statistical analysis*

Descriptive statistics were used to summarize characteristics of the study population. Adequacy of dietary intake was tested by comparing energy and protein intake with the respective energy or protein requirements in a paired samples t-test. All outcomes were analysed as between-group differences in outcomes using intention-to-treat ANCOVA, adjusted for baseline values of the outcome and stratification factors (i.e., sex, hospital and time since surgery). Standardized effect sizes (ESs) were calculated. Analyses were performed using IBM SPSS Statistics 25.0. All tests were two-tailed and the significance level was set at p<0.05.

**References**

1. Kruizenga HM, Hofsteenge GH, Weijs PJM. Predicting resting energy expenditure in underweight, normal weight, overweight, and obese adult hospital patients. *Nutr Metab (Lond)*. 2016;13(1):85. doi:10.1186/s12986-016-0145-3

2. Kruizenga HM, Beijer S, Huisman-de Waal G, et al. *Richtlijn Ondervoeding: Herkenning, Diagnosestelling En Behandeling van Ondervoeding Bij Volwassenen*.; 2019.

3. National Collaborating Centre for Acute Care (UK). *Nutrition Support for Adults: Oral Nutrition Support, Enteral Tube Feeding and Parenteral Nutrition.*; 2006.

4. Kruizenga HM, Beijer S, Huisman-de Waal G, et al. *Richtlijn Ondervoeding: Herkenning, Diagnosestelling En Behandeling van Ondervoeding Bij Volwassenen*.; 2019.

5. Muscaritoli M, Arends J, Bachmann P, et al. ESPEN practical guideline: Clinical Nutrition in cancer. *Clin Nutr*. 2021;40(5):2898-2913. doi:10.1016/j.clnu.2021.02.005

6. Ishibashi N, Plank LD, Sando K, Hill GL. Optimal protein requirements during the first 2 weeks after the onset of critical  illness. *Crit Care Med*. 1998;26(9):1529-1535. doi:10.1097/00003246-199809000-00020

7. Weijs PJM, Sauerwein HP, Kondrup J. Protein recommendations in the ICU: g protein/kg body weight - which body weight  for underweight and obese patients? *Clin Nutr*. 2012;31(5):774-775. doi:10.1016/j.clnu.2012.04.007

8. Dekker IM, van Rijssen NM, Verreijen A, et al. Calculation of protein requirements; a comparison of calculations based on  bodyweight and fat free mass. *Clin Nutr ESPEN*. 2022;48:378-385. doi:10.1016/j.clnesp.2022.01.014

9. Kyle UG, Genton L, Karsegard L, Slosman DO, Pichard C. Single prediction equation for bioelectrical impedance analysis in adults aged 20--94 years. *Nutrition*. 2001;17(3):248-253. doi:10.1016/s0899-9007(00)00553-0

10. Schutz Y, Kyle UUG, Pichard C. Fat-free mass index and fat mass index percentiles in caucasians aged 18-98 y. *Int J Obes*. 2002;26(7):953-960. doi:10.1038/sj.ijo.0802037

11. Weir JB de V. New methods for calculating metabolic rate with special reference to protein metabolism. *J Physiol*. 1949;109(1-2):1-9. doi:10.1113/jphysiol.1949.sp004363

12. Boothby WM, Sandiford I. Summary of the Basal Metabolism Data on 8,614 Subjects With Especial Reference To the Normal Standards for the Estimation of the Basal Metabolic Rate. *Journal of Biological Chemistry*. 1922;54(4):783-803. doi:10.1016/s0021-9258(18)85715-5

13. Purcell SA, Elliott SA, Baracos VE, Chu QS, Prado CM. Key determinants of energy expenditure in cancer and implications for clinical practice. *Eur J Clin Nutr*. 2016;70(11):1230-1238. doi:10.1038/ejcn.2016.96

14. Soares MJ, Müller MJ. Resting energy expenditure and body composition: critical aspects for clinical nutrition. *Eur J Clin Nutr*. 2018;72(9):1208-1214. doi:10.1038/s41430-018-0220-0

15. Gabrielson DK, Scaffidi D, Leung E, et al. Use of an abridged scored Patient-Generated Subjective Global Assessment (abPG-SGA) as a nutritional screening tool for cancer patients in an outpatient setting. *Nutr Cancer*. 2013;65(2):234-239. doi:10.1080/01635581.2013.755554

16. Abbott J, Teleni L, McKavanagh D, Watson J, McCarthy AL, Isenring E. Patient-Generated Subjective Global Assessment Short Form (PG-SGA SF) is a valid  screening tool in chemotherapy outpatients. *Support Care Cancer*. 2016;24(9):3883-3887. doi:10.1007/s00520-016-3196-0

17. Jager-Wittenaar H, Ottery FD. Assessing nutritional status in cancer: role of the Patient-Generated Subjective Global Assessment. *Curr Opin Clin Nutr Metab Care*. 2017;20(5):322-329. doi:10.1097/mco.0000000000000389

| **Table S1.** Baseline characteristics of all participants in the PERFECT study and participants in the dietetic study. | | | | | | |
| --- | --- | --- | --- | --- | --- | --- |
|  |  | **Participants PERFECT study** | |  | **Participants Dietetic study** | |
|  |  | **Intervention *n = 61*** | **Usual Care *n = 59*** |  | **Intervention *n = 19*** | **Usual Care *n = 18*** |
| **Age (years)**† |  | 64.3 (7.8) | 63.1 (8.5) |  | 63.6 (8.6) | 58.3 (7.6) |
| **Sex**  Male  Female |  | 52 (85.2)  9 (14.8) | 52 (88.1)  7 (11.9) |  | 16 (84.2)  3 (15.8) | 16 (88.9)  2 (11.1) |
| **Educational Level**  Low  Middle  High |  | 16 (26.2)  29 (47.5)  16 (26.2) | 16 (27.1)  30 (50.8)  13 (22.0) |  | 5 (26.3)  7 (36.8)  7 (36.8) | 3 (16.7)  10 (55.6)  5 (27.8) |
| **Marital Status**  Couple  Single  Widow |  | 55 (90.2)  5 (8.2)  1 (1.6) | 52 (88.1)  7 (11.9)  0 (0.0) |  | 17 (89.5)  2 (10.5)  0 (0.0) | 14 (77.8)  4 (22.2)  0 (0.0) |
| **Work Status**  Paid work  Sick leave  No paid work/retired |  | 26 (42.6)  18 (69.2)  35 (57.4) | 21 (35.6)  14 (66.7)  38 (64.4) |  | 9 (47.4)  6 (66.7)  10 (52.6) | 10 (55.6)  7 (70.0)  8 (44.4) |
| **Smoking**  Current  Never  Former |  | 3 (4.9)  12 (19.7)  46 (75.4) | 7 (11.9)  12 (20.3)  40 (67.8) |  | 0 (0.0)  13 (68.4)  6 (31.6) | 0 (0.0)  13 (72.2)  5 (27.8) |
| **Cancer type**  Adenocarcinoma  Squamous cell carcinoma  Adenosquamous  Other |  | 49 (80.3)  9 (14.8)  0  3 (4.9) | 43 (72.9)  11 (18.6)  1 (1.7)  4 (6.8) |  | 17 (89.5)  2 (10.5)  0 (0.0)  0 (0.0) | 14 (77.8)  2 (11.1)  0 (0.0)  2 (11.1) |
| **Tumor stage**  I  II  III |  | 10 (16.4)  16 (26.2)  34 (55.7) | 6 (10.2)  23 (39.0)  30 (50.8) |  | 2 (10.5)  4 (21.1)  13 (68.4) | 3 (16.7)  5 (27.8)  10 (55.8) |
| **Comorbidities**  Yes  No |  | 21 (34.4)  40 (65.6) | 28 (47.5)  31(52.5) |  | 7 (36.8)  12 (63.2) | 8 (44.4)  10 (55.6) |
| **Type of surgery**  Open oesophagectomy  Minimally-invasive oesophagectomy |  | 5 (8.2)  56 (91.8) | 6 (10.2)  53 (89.9) |  | 3 (15.8)  16 (84.2) | 3 (16.7)  15 (83.3) |
| **Complications after surgery**  Yes  No |  | 41 (67.2)  20 (32.8) | 35 (59.3)  24 (40.7) |  | 13 (69.4)  6 (31.6) | 12 (66.7)  6 (33.3) |
| **Time since surgery** (median (IQR))  0-5 months  6-12 months |  | 3.0 (2.0-6.5)  41 (67.2)  20 (32.8) | 4.0 (2.0-7.0)  40 (67.8)  19 (32.2) |  | 3.0 (1.0-10.0)  11 (57.9)  8 (42.1) | 3.0 (2.0-8.0)  12 (66.7)  6 (33.3) |
| **(Neo)adjuvant treatment**  Chemotherapy  Chemoradio-therapy  Chemoradio-therapy + immunotherapy  No (neo)adjuvant treatment |  | 1 (1.6)  50 (81.9)  1 (1.6)  9 (14.8) | 6 (10.2)  46 (78.0)  1 (1.7)  6 (10.2) |  | 0 (0.0)  16 (84.2)  0 (0.0)  3 (15.8) | 2 (11.1)  13 (72.2)  0 (0.0)  3 (16.7) |
| ٭Values in parentheses are percentages; †values are mean (s.d.). | | | | | | |

| **Table S2.** Nutritional characteristics of all participants in the PERFECT study and participants in the dietetic study. | | | | | | | | | | | | | |
| --- | --- | --- | --- | --- | --- | --- | --- | --- | --- | --- | --- | --- | --- |
|  |  | **Baseline** | | | | |  | **12 weeks (post-intervention)** | | | | | |
|  |  | **Participants PERFECT study** | |  | **Participants Dietetic study** | |  | **Participants PERFECT study** | |  | **Participants Dietetic study** | | |
|  |  | **Intervention *n = 61*** | **Usual Care *n = 59*** |  | **Intervention *n = 19*** | **Usual Care *n = 18*** |  | **Intervention *n = 61*** | **Usual Care *n = 59*** |  | **Intervention *n = 19*** | | **Usual Care *n = 18*** |
| **BMI (kg/m^2^)**† |  | 24.8 (3.2) | 25.0 (3.8) |  | 24.5 (3.3) | 24.6 (3.1) |  | 24.6 (3.1) | 24.8 (3.6) |  | 24.4 (2.9) | 24.5 (3.7) | |
| **FFMI (kg/m²)**†  Low FFMI‡ |  | - | - |  | 18.2 (2.1)  2 (10.5) | 18.2 (2.1)  3 (16.7) |  | -  - | -  - |  | 18.5 (2.2)  2 (10.5) | 18.2 (2.3)  3 (16.7) | |
| **PG-SGA SF score**  Weight  Food intake  Symptoms  Activities and function |  | 7.4 (5.2)  1.0 (1.4)  1.2 (0.6)  4.2 (4.1)  1.0 (0.8) | 7.4 (5.2)  0.6 (1.3)  1.2 (0.5)  4.4 (4.0)  1.1 (0.8) |  | 8.6 (4.7)  1.2 (1.5)  1.2 (0.5)  5.0 (4.7)  1.3 (0.9) | 6.1 (4.7)  0.3 (1.0)  1.2 (0.5)  3.5 (4.0)  1.2 (0.9) |  | 5.6 (4.4)  0.7 (1.2)  1.1 (0.6)  3.1 (3.2)  0.7 (0.9) | 6.6 (5.8)  0.5 (1.0)  1.1 (0.5)  4.0 (5.0)  0.8 (0.9) |  | 6.1 (4.5)  0.9 (1.5)  1.0 (0.4)  3.5 (2.8)  0.9 (1.2) | 7.8 (6.8)  0.6 (1.2)  1.1 (0.4)  1.2 (0.5)  1.1 (1.0) | |
| **Malnutrition Risk**‡‡  Low risk  Medium risk  High risk |  | 16 (26.2)  23 (37.7)  21 (34.4) | 15 (25.4)  22 (37.3)  18 (30.5) |  | 3 (15.8)  6 (31.6)  10 (52.6) | 8 (44.4)  4 (22.2)  6 (33.3) |  | 20 (32.8)  18 (29.5)  11 (18.0) | 21 (35.6)  18 (30.5)  14 (23.7) |  | 5 (26.3)  6 (31.6)  4 (21.1) | 7 (38.9)  6 (33.3)  5 (27.8) | |
| **Nutritional Status**  Well-nourished  Moderate / suspected   malnutrition  Severely malnourished |  | -  -  - | -  -  - |  | 8 (42.1)  11 (57.9)  0 (0.0) | 11 (61.1)  6 (33.3)  1 (5.6) |  | -  -  - | -  -  - |  | 10 (52.6)  4 (21.1)  2 (10.5) | 12 (66.7)  1 (5.6)  2 (11.1) | |
| **Medical nutrition therapy**  Oral nutritional supplements  Enteral tube feeding   - Total   - Supplemental |  | 19 (31.1)  3 (4.9)  3 (4.9) | 11 (18.6)  1 (1.7)  3 (5.1) |  | 7 (36.8)  2 (10.5)  2 (10.5) | 4 (22.2)  0 (0.0)  0 (0.0) |  | 10 (16.4)  0 (0.0)  0 (0.0) | 8 (13.6)  1 (1.7)  0 (0.0) |  | 1 (5.3)  0 (0.0)  0 (0.0) | 2 (11.1)  0 (0.0)  0 (0.0) | |
| **mREE compared with pREE**‡‡‡  Hypometabolism Normometabolism  Hypermetabolism |  | -  -  - | -  -  - |  | 1 (5.3)  14 (73.7)  4 (21) | 0 (0.0)  14 (77.8)  4 (22.2) |  | -  -  - | -  -  - |  | 0 (0.0)  11 (57.9)  5 (26.3) | 1 (5.6)  14 (77.8)  1 (5.6) | |
| *Values in parentheses are percentages; †values are mean (s.d.). ‡A low FFM Index was defined as <16.7 kg/m² for men and <14.6 kg/m² for women. ‡‡The risk for malnutrition was assessed using the PG-SGA SF, with a score lower than 4 indicating a low risk, 4-8 medium risk, and greater than 8 high risk. ‡‡‡Hypometabolism was defined as measured REE (mREE) <90% of predicted REE (pREE), normometabolic as mREE between 90% and 110% of the pREE, and hypermetabolism as mREE >110% of pREE. | | | | | | | | | | | | | |

| **Table S3.** Difference between energy intake and measured and estimated total energy expenditure. | | | | |
| --- | --- | --- | --- | --- |
| **All PERFECT participants** | | Energy intake (kcal)  Mean (SD) | Difference between intake and mTEE (kcal)  Mean (95% CI) | Difference between intake and pTEE (kcal)  Mean (95% CI) |
| Baseline | Whole group | 2407 (627) | - | -21 (-133; 91) |
|  | EX | 2451 (696) | - | 31 (-143; 195) |
|  | UC | 2359 (545) | - | -78 (-232; 77) |
| 12 weeks | Whole group | 2473 (621) | - | 39 (-76; 155) |
|  | EX | 2480 (694) | - | 96 (-87; 279) |
|  | UC | 2466 (538) | - | -19 (-164; 125) |
| **Dietetic subgroup** | | | | |
| Baseline | Whole group | 2322 (681) | -185 (-394; 23) | -81 (-296; 134) |
|  | EX | 2397 (759) | 1.6 (-293; 296) | 64 (-261; 388) |
|  | UC | 2238 (594) | -394 (-686; -102)* | -243 (-534; 49) |
| 12 weeks | Whole group | 2269 (602) | -231 (-415; -47)* | -136 (-317; 45) |
|  | EX | 2243 (585) | -242 (-491; 8) | -76 (-324; 172) |
|  | UC | 2296 (637) | -220 (-520; 80) | -200 (-491; 91) |

* Statistically significant (p < 0.05)

| **Table S4.** Adequacy of energy intake. | | | |
| --- | --- | --- | --- |
| **All PERFECT participants** | | Adequate energy intake based on pTEE  *n* (%) | Adequate energy intake based on mTEE  *n* (%) |
| Baseline | Whole group | 74 (63.2) | - |
|  | EX | 38 (62.3) | - |
|  | UC | 36 (64.3) | - |
| 12 weeks | Whole group | 70 (66.0) | - |
|  | EX | 34 (63.0) | - |
|  | UC | 36 (69.2) | - |
| **Dietetic subgroup** | | | |
| Baseline | Whole group | 22 (61.1) | 20 (55.6) |
|  | EX | 12 (63.2) | 10 (52.6) |
|  | UC | 10 (58.8) | 10 (58.8) |
| 12 weeks | Whole group | 18 (54.5) | 14 (43.8) |
|  | EX | 9 (52.9) | 7 (43.8) |
|  | UC | 9 (56.3) | 7 (43.8) |

| **Table S5.** Difference between protein intake and protein recommendations (g/kg and g/FFM). | | | | |
| --- | --- | --- | --- | --- |
| **All PERFECT participants** | | Protein intake (g)  Mean (SD) | Difference between intake and recommendation g/kg (g)  Mean (95% CI) | Difference between intake and recommendation g/FFM (g)  Mean (95% CI) |
| Baseline | Whole group | 93.79 (27.82) | -19.18 (-24.36; -14.00)* | - |
|  | EX | 95.29 (28.75) | -16.47 (-23.76; -9.18)* | - |
|  | UC | 92.16 (26.94) | -22.14 (-29.66; -14.62)* | - |
| 12 weeks | Whole group | 93.57 [73.11; 111.94] | -20.43 [-5.35]* | - |
|  | EX | 92.00 [72.62; 109.44] | -22.56 [-3.25]* | - |
|  | UC | 95.12 [73.78; 113.61] | -21.56 [-4.32]* | - |
| **Dietetic subgroup** | | | | |
| Baseline | Whole group | 91.68 (24.78) | -20.03 (-29.63; -10.44)* | -14.76 (-23.50; -6.02)* |
|  | EX | 94.81 (23.47) | -13.36 (-25.90; -0.82)* | -9.41 (-21.44; 2.62) |
|  | UC | 88.17 (26.44) | -27.49 (-42.78; -12.20)* | -20.75 (-34.21; -7.28)* |
| 12 weeks | Whole group | 89.74 (28.81) | -23.03 (-33.60; -12.45)* | -18.57 (-28.15; -8.99)* |
|  | EX | 83.83 (23.47) | -26.06 (-37.85; -14.27)* | -22.78 (-33.68; -11.89)* |
|  | UC | 95.65 (33.01) | -19.99 (-38.98; -1.00)* | -14.35 (-31.24; 2.54) |

* Statistically significant (p < 0.05)

| **Table S6.** Adequacy of protein intake. | | | |
| --- | --- | --- | --- |
| **All PERFECT participants** | | Adequate protein intake based on recommendation g/kg *n* (%) | Adequate protein intake based on recommendation g/FFM  *n* (%) |
| Baseline | Whole group | 44 (37.6) | - |
|  | EX | 28 (45.9) | - |
|  | UC | 16 (28.6) | - |
| 12 weeks | Whole group | 38 (35.8) | - |
|  | EX | 21 (38.9) | - |
|  | UC | 17 (32.7) | - |
| **Add-on subgroup** | | | |
| Baseline | Whole group | 12 (33.3) | 18 (50.0) |
|  | EX | 7 (36.8) | 11 (57.9) |
|  | UC | 5 (29.4) | 7 (41.2) |
| 12 weeks | Whole group | 13 (39.4) | 11 (34.4) |
|  | EX | 6 (35.3) | 4 (25.0) |
|  | UC | 7 (43.8) | 7 (43.8) |

**
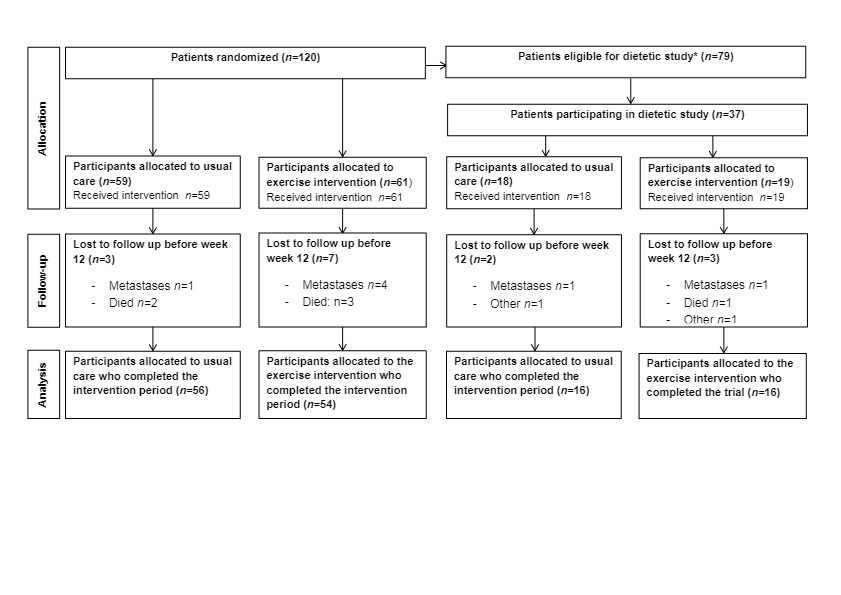
**

**Figure S1.** Flow of participants in the PERFECT study and dietetic study.

*Only participants from UMC Utrecht, St. Antonius Hospital, IJsselland Hospital, Radboud University Medical Centre, Amsterdam UMC (locations: VUMC and AMC) and Erasmus MC, were eligible for participation in the dietetic study.
